# An ultrasensitive and specific CRISPR-Cas13a-mediated point-of-care assay for monkeypox detection and PCR-based clade detection

**DOI:** 10.1186/s40249-025-01325-5

**Published:** 2025-06-23

**Authors:** Qin Zhang, Yan Yu, Bin Yin, Liang Xu, Hui Chen, Runjie Qiao, Ang Chen, Na Zhu, Xuping Wu

**Affiliations:** 1 The Precision Medical Center, The Second Hospital of Nanjing, Nanjing University of Chinese Medicine, Nanjing, 210003 Jiangsu China; 2 Suzhou TianLong Biotechnology, Suzhou, 225300 Jiangsu China

**Keywords:** Monkeypox, CRISPR-Cas13a system, Point-of-care assay, Lateral flow strip, MPXV clade detection

## Abstract

**Background:**

The rapid increase in the number of monkeypox cases poses a considerable threat to the international community, necessitating sensitive, fast, and available diagnostic methods. Therefore, the objective of this study was to develop a rapid, sensitive and simple method with high clinical applicability.

**Methods:**

We developed a simple, rapid point-of-care assay to detect monkeypox virus (MPXV) using multienzyme isothermal rapid amplification (MIRA) coupled with the clustered regularly interspaced short palindromic repeats (CRISPR)-Cas13a system. The detection system was optimized by synthesizing plasmids, and the detection sensitivity was explored by the continuous dilution of the plasmid. We validated the accuracy of this assay on 202 clinical MPXV samples and 104 interference samples through the kappa test. The visual interpretation of the results was realized by combining the assay with lateral flow strips. In addition, we developed a PCR-based method to identify MPXV Clades I and II, and the accuracy was tested through a kappa test on 202 clinical monkeypox samples and 104 interference samples.

**Results:**

Our assay achieved an analytical sensitivity of 14.4 copies/ml and high selectivity, as it differentiated MPXV from three other *Orthopoxvirus* species. The clinical testing results for 202 monkeypox samples and 104 interference samples demonstrated 100% sensitivity and specificity. Compared with quantitative PCR (qPCR), three samples tested as positive using our assay, which showed that the performance of this assay was superior to that of the qPCR assay. Combined with lateral flow strips, its availability and simplicity provide an alternative point-of-care diagnostic method for MPXV testing in remote settings and resource-poor areas. The results of 32 clinical samples showed that lateral flow strips had a high detection sensitivity and could identify samples with Ct value of 39 as positive. The clade identification assay detected as few as 200 copies/ml within 40 min and no cross-reaction was observed between Clades I and II. The clinical samples tested were all Clade II, which was consistent with the circulating clade in the Chinese mainland.

**Conclusions:**

The MIRA-CRISPR-Cas13a-MPXV system offers a rapid, sensitive and specific approach for monkeypox diagnosis, with significance for monitoring monkeypox epidemics. The clade identification assay based on PCR could accurately distinguish Clade I from Clade II within 40 min and can be implemented for high-throughput operation.

**Graphical abstract:**

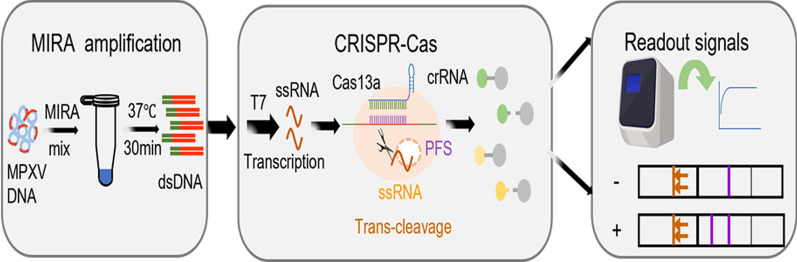

**Supplementary Information:**

The online version contains supplementary material available at 10.1186/s40249-025-01325-5.

## Background

Monkeypox is a zoonotic disease caused by a monkeypox virus (MPXV) infection that leads to a rash similar to that caused by smallpox [1, 2]. MPXV is a double-stranded DNA virus that belongs to the genus *Orthopoxvirus* (OPV) of the Poxviridae family and includes cowpox virus, variola virus and vaccinia virus [3–5]. MPXV is transmitted through close contact with infected animals, large respiratory droplets, skin lesions, maternal-infant transmission and sexual transmission [6, 7]. Based on the clinical manifestations and genomic sequences, MPXV can be classified into Central African (Clade I) and West African (Clade II) clades, with the former causing more serious diseases and the latter being less virulent but more widely spread [8–10]. The incubation period of monkeypox infection is usually 5–21 days, and the common clinical symptoms include fever, headache, myalgia, swollen lymph nodes and a characteristic rash [3, 8, 11]. Several studies have shown that smallpox vaccination provides some protection against MPXV infection because of the high degree of genomic homology between the two viruses [12–14]. However, since the termination of the smallpox vaccination regime after smallpox eradication worldwide in the 1980s, many unvaccinated people are vulnerable to MPXV infection without a previous exposure and the development of immunity [15–18]. Unfortunately, specific anti-MPXV drugs are not yet available, which increases the difficulty of preventing and controlling MPXV epidemics [16]. Thus, the development of precise and rapid diagnostic methods is crucial for the surveillance of MPXV.

Currently, MPXV infection is diagnosed based on the suspected epidemiological situation and clinical manifestations and is confirmed by fluorescent quantitative PCR [19, 20]. In Africa, where the epidemic is raging, most cases are not laboratory confirmed due to a lack of diagnostic infrastructure and poor medical conditions [21, 22]. Serological tests based on enzyme-linked immunosorbent assays, including antigen and antibody tests, are also commonly used in laboratory tests because of their speed and simplicity [23]. However, these methods may not provide a specific diagnosis because of the high degree of molecular similarity among numerous OPVs, leading to false-positive results [3, 24]. Moreover, few reports are available on the detection of MPXV clades [25–27]. A valuable lesson learned from effective COVID-19 mitigation strategies is that a rapid and accurate diagnosis, as well as clade identification, are critical for the control and timely treatment of MPXV.

Recently, the clustered regularly interspaced short palindromic repeats (CRISPR)-Cas-based system has shown promise as a viable alternative in rural clinics in low-income countries where fluorescent quantitative PCR machines are not available. In previous COVID-19 outbreaks, the CRISPR-Cas system was reported to be successfully used in COVID-19 diagnostic studies [28, 29]. The CRISPR-Cas system is perfectly compatible with the point-of-care testing (POCT) because of its superior detection performance and low equipment requirements. The lateral flow assay is an ideal signal reporter for capturing CRISPR-Cas cleavage products because of its low cost, easy operation, and excellent stability. Here, we report the use of CRISPR-Cas13a in combination with a multienzyme isothermal rapid amplification (MIRA) MPXV assay. This study aims to fill the gap imposed by the unavailability of testing due to the use of expensive reagents and precision instruments in remote areas. When the MIRA reaction product was added to the CRISPR-Cas13a reaction mixture, LwaCas13a induced powerful nonspecific trans-cleavage of the single-stranded RNA guided by a specific CRISPR RNA (crRNA), which could be collected and analysed by fluorescence detectors or lateral flow strips. This assay has the potential to be utilized in remote areas and could greatly enhance the diagnostic capabilities of MPXV. In total, 202 clinical samples and 104 interference samples were tested to validate the clinical feasibility of this assay. In addition, we developed a fluorescent PCR-based approach to identify different MPXV clades through the design of specific primers and probes targeting the variable region of the *C3L* gene in Clades I and II. In summary, this study aimed to develop an assay with good sensitivity, specificity and availability for point-of-care detection of MPXV in remote areas.

## Methods

### Reagents and materials

In this study, we targeted the highly conserved MPXV *F3L* gene to design primers. The MPXV *F3L* gene sequence was derived from the reference sequence (GenBank: NC_063383.1) in NCBI uploaded by the United States in May 2022. We designed four forward primers and four reverse primers, as shown in Additional file 1: Table S1, and screened out the best group by 2% agarose gel electrophoresis (AGE). We added the T7 promoter sequence to the 5' end of the forward primers for the MIRA to facilitate reverse transcription by T7 RNA polymerase. According to the AGE results of MIRA, we designed three candidate 28-nt guide sequences and screened the optimal crRNA using a fluorescence assay. The oligonucleotides used in the MIRA-CRISPR-Cas13a system were synthesized and purified by General Biol (Anhui, China) and are listed in Additional file 1: Table S1. The primers and probes used for clade identification were synthesized and purified by Sangon Biotech (Shanghai, China) and are listed in Additional file 1: Table S2. The MPXV, cowpox, vaccinia, and variola *F3L* gene fragments used in this study were synthesized by General Biol (Anhui, China), cloned and inserted into the pUC57 vector and are listed in Additional file 1: Table S3. The above sequences of the three *F3L* gene fragments from viruses other than MPXV were obtained from the study by Wang et al. [30]. The MPXV, Clade I, Clade II, and GAPDH genes used in clade identification were synthesized by Sangon Biotech (Shanghai, China) and are listed in Additional file 1: Table S4. The number of plasmid copies was calculated using the following formula: copies/ml = 6.02 × 10^23^ × concentration (g/ml) / (sequence length × 660).

The MIRA kit (WLB8201KIT) was obtained from Amp-Future Biotech (Jiangsu, China). The reaction buffer (10 ×), LwaCas13a and its dilution buffer were purchased from EZAssay Biotech (Guangdong, China). The rNTP mixture was purchased from New England Biolabs (Ipswich, MA, USA). The RNase inhibitor and T7 RNA polymerase were purchased from Takara (Beijing, China). The nucleic acid test strips ( MGHD-1, Cat.No.: MGDS) were purchased from Milenia Biotec (Gießen, Germany).

### Collection and validation of clinical samples

This study was approved by the Medical Ethics Committee of the Second Hospital of Nanjing (2024-LS-ky-113). Positive clinical samples (total, *n* = 179; rash fluid swabs, *n* = 51; throat swabs, *n* = 52; anal swabs, *n* = 46; serum, *n* = 9; plasma, *n* = 21) were obtained from patients diagnosed with MPXV infections based on the clinical symptoms, laboratory tests and epidemiological evidence (detailed information is provided in Additional file 1: Table S5). Negative clinical samples (total, *n* = 23; rash fluid swabs, *n* = 4; throat swabs, *n* = 9; anal swabs, *n* = 1; serum, *n* = 1; and plasma, *n* = 8;) were obtained from MPXV-infected or suspected MPXV-infected individuals, If the samples were obtained from individuals with confirmed or suspected monkeypox infections, they should be classified as "positive" or "unconfirmed," not "negative." as shown in Additional file 1: Table S5. Interfering samples (*n* = 104) including Severe fever with thrombocytopenia syndrome virus (SFTSV), dengue fever virus (DFV), influenza A virus (INF-A), influenza B virus (INF-B), SARS-CoV-2, hepatitis B virus (HBV), hepatitis C virus (HCV), human immunodeficiency virus (HIV), Epstein-Barr virus (EBV), cytomegalovirus (CMV), human papillomavirus (HPV), varicella-zoster virus (VZV), *Escherichia coli*, *Klebsiella pneumoniae* (KP), *Pseudomonas aeruginosa* (PA), *Enterobacter cloacae* (EC), *Enterococcus faecalis* (EF), *Bordetella pertussis* (BP), *Mycobacterium tuberculosis* (MTB) and nontuberculous mycobacteria (NTM) were collected from the Second Hospital of Nanjing (detailed information is provided in Additional file 1: Table S6). All the clinical samples were collected by professional clinicians and stored in disposable virus sampling tubes. Considering the long storage time of the collected samples and whether the viral load changed, we conducted qPCR detection of the collected samples again. Nucleic acids were extracted from clinical samples (rash fluid swabs, throat swabs, anal swabs, serum, and plasma) using a fully automatic nucleic acid extractor (Jiangsu Bioperfectus Technologies). Real-time qPCR assays for MPXV were performed using a clinically validated kit approved by the Conformite European certification and National Medical Products Administration (DAAN gene, Guangdong, China) on a QuantStudio^TM^5 fluorescence qPCR instrument (Thermo Fisher Scientific, the United States) according to the instructions, with cycle threshold (Ct) values below or equal to 40 considered positive. The running procedure was as follows: 50 °C for 2 min, 1 cycle; 95 °C for 5 min, 1 cycle; and 95 °C for 5 s, 60 °C for 35 s (fluorescence detection), 45 cycles. Other interference samples were determined through laboratory standard testing and analysis methods and detected using the aforementioned kit to ensure the accuracy of the results.

### MIRA system

We performed a MIRA reaction to amplify the MPXV DNA, followed by 2% AGE to identify the best combinations of the sixteen pairs of primers. A total of 50 µl of master mix was prepared with 2 µl of forwards and 2 µl of reverse primers (10 µmol/L), 29.4 µl of rehydration buffer, 5 µl of MPXV *F3L* gene DNA template, and 9.1 µl of DNase/RNase-free water. Magnesium acetate (2.5 µl, 280 mmol/L) was finally added to initiate the MIRA reaction and was incubated at 37 °C for 30 min. Then, 10 µl of the DNA amplicons of the MIRA reaction was mixed with 2 µl of 6 × DNA loading dye for 5 min at 56 °C and 2% AGE was performed. The gel image was captured by a gel imaging system (Tanon, Shanghai, China) and Tanon image analysis software was used to process the images.

### The CRISPR-Cas13a system

According to the MIRA product sequences, we designed three candidate crRNAs that avoided forward and reverse primer binding regions. The CRISPR-Cas13a reaction for crRNA screening was performed under the following conditions: a total of 20 µl of mixture contained 1 µl of MIRA product, 1 µl of crRNA (0.5 µmol/L), 2 µl of LwaCas13a (1 µmol/L), 0.8 µl of rNTPs (25 nmol/L), 10.6 µl of DNase/RNase-free water, 0.6 µl of T7 RNA polymerase (50 U/µl), 1 µl of RNase inhibitor (40 U/µl), 2 µl of reaction buffer (10 ×) and 1 µl of reporter (2 µmol/L). The running procedure was performed on a SLAN-96S instrument (Shanghai, China) as follows: 37 °C for 30 s, 60 cycles (fluorescence detection).

After selecting the optimal crRNA (crRNA-3), we optimized several essential components of the assay system, including LwaCas13a, crRNA and the reporter. LwaCas13a concentrations ranged from 1 to 10 µmol/L, crRNA concentrations ranged from 0.25 to 5 µmol/L, and reporter concentrations ranged from 2 to 20 µmol/L. Moreover, we performed the following tests to verify that several essential components of the CRISPR-Cas13a system are indispensable, each tube reaction lacked a component, such as T7 RNA polymerase, LwaCas13a, crRNA, reporter, or the MIRA product.

### Evaluation of MIRA-CRISPR-Cas13a-MPXV detection sensitivity and specificity

The synthetic MPXV *F3L* gene plasmid was continuously diluted, and this process was repeated three times under optimal conditions to explore the minimum detection sensitivity of this assay. The cowpox, vaccinia, and variola *F3L* gene fragments were synthesized to evaluate specificity. We further verified the specific performance of the MIRA-CRISPR-Cas13a-MPXV fluorescence assay in clinical samples, by performing analytical specificity experiments on a range of pathogens including but not limited to the varicella zoster virus. The assay was performed as previously described.

### Application of the MIRA-CRISPR-Cas13a-MPXV assay in clinical samples

The application of the MIRA-CRISPR-Cas13a-MPXV fluorescence assay in clinical samples was analysed using 202 clinical samples obtained from the Second Hospital of Nanjing. The clinical samples included several types of monkeypox samples. The assay was performed under the following conditions: a mixture of 1 µl of MIRA product, 1 µl of crRNA (0.5 µmol/L), 2 µl of LwaCas13a (1 µmol/L), 0.8 µl of the rNTP mixture (25 nmol/L), 10.6 µl of DNase/RNase-free water, 0.6 µl of T7 RNA polymerase (50 U/µl), 1 µl of RNase inhibitor (40 U/µl), 2 µl of reaction buffer (10 ×) and 1 µl of the FAM-BHQ1(FQ) reporter (10 µmol/L). The mixture was analysed using the SLAN-96S instrument. The running procedure was as follows: 37 °C for 30 s and 60 cycles (fluorescence detection).

The reaction system of the MIRA-CRISPR-Cas13a-MPXV lateral flow strip assay was similar to that of the fluorescence assay described above, except that the FQ reporter was replaced with a FITC-biotin (FB) reporter. After an incubation at 37 °C for 30 min, 10 µl of product and 100 µl of detection buffer were mixed and applied to the lateral flow strip. The result was visualized after 5 min of incubation at room temperature.

### Identification of MPXV clades

A fluorescent PCR-based clade assay was developed to identify Clades I and II of MPXV. A total of 25 µl of the mixture was analysed using a QuantStudio^TM^5 fluorescence quantitative PCR instrument. The running procedure was as follows: 95 °C for 3 min, 1 cycle; and 95 °C for 5 s, and 60 °C for 20 s (fluorescence detection), 45 cycles. We synthesized monkeypox *F3L* gene plasmids, Clade I *C3L* gene plasmids and Clade II del*C3L* gene plasmids (shown in Additional file 1: Table S4) and diluted the three plasmids to 500, 200, 150 and 100 copies/ml, respectively, to explore the minimum limit of detection (LOD) of this method. The detection was repeated 20 times for each concentration. After the minimum LOD was calculated, three plasmids encoding the monkeypox *F3L* gene, Clade I *C3L* gene and Clade II del*C3L* gene were prepared to verify the minimum LOD concentration. The analysis of each sample containing the LOD concentration was repeated 20 times, and the positive detection rate was required to be ≥ 95%. The cross-specific detection of Clades I and II was verified by mutual detection. In addition, we detected cowpox, vaccinia, and variola viruses to verify the specificity of this assay among OPVs. We further verified the specificity of this assay in clinical samples by detecting 202 clinical samples and 104 interference samples.

### Data analysis

Continuous variables with a normal distribution are presented as the means ± standard deviations (SDs). One-way ANOVA in GraphPad Prism software version 9.5 (Boston, the United States) was conducted to determine the sensitivity of the *F3L* gene, and the results are presented as means ± SDs. A probit regression analysis in IBM SPSS software version 26 (SPSS Inc., Chicago, the United States) was performed to determine the LOD for clade identification. The concentration that could be detected with a probability of 95% was defined as the minimum LOD. The consistency of the results from the different methods was determined by calculating Cohen’s kappa coefficient using IBM SPSS software version 26 (SPSS Inc., Chicago, the United States). The kappa value was interpreted as follows: < 0.4, limited; 0.41–0.75, moderate; and ≥ 0.75, excellent. For all analyses, *P* < 0.05 was considered statistically significant. Significance is indicated by asterisks: ∗ , *P* < 0.05; ∗ ∗ , *P* < 0.01; ∗ ∗ ∗ , *P* < 0.001; ∗ ∗ ∗ ∗ , *P* < 0.0001; and ns, not significant.

## Results

### Development and verification of the MIRA-CRISPR-Cas13a-MPXV system

**Fig. 1:**
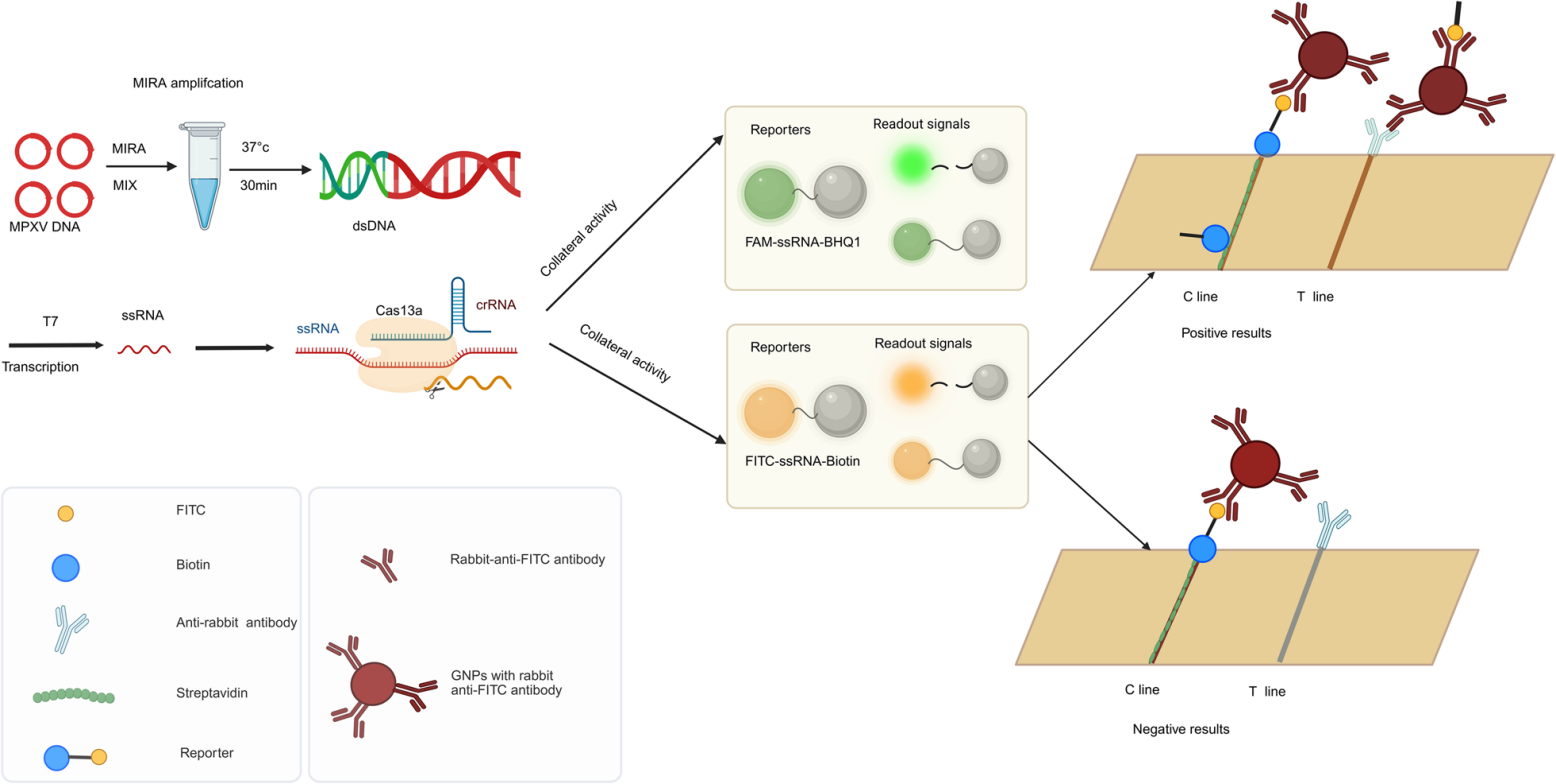
The principle of MIRA-CRISPR-Cas13a proposed in this study. MIRA for target template amplification; T7 transcription for dsDNA to target ssRNA; CRISPR-Cas13a for reporter cleavage; fluorescence assay and lateral flow strip assay for detection signal output

As shown in Fig. 1, the extracted nucleic acid was first reacted in the MIRA solution to obtain sufficient amplicons. Then, the amplified product was transcribed into ssRNA by T7 RNA polymerase in the CRISPR-Cas13a system, and the trans-cleavage activity of LwaCas13a was recognized and activated by the complex of ssRNA, crRNA and LwaCas13a. The reporters in the reaction mixture were subsequently cut. Finally, the signal output presented two modes based on the different reporters: a fluorescence assay for FQ (6-FAM at the 5' end and BHQ1 at the 3' end) and a lateral flow strip assay for FB (FITC at the 5' end and biotin at the 3' end). The fluorescence could be observed by a simple fluorescence detector and the lateral flow strip could be observed by the naked eye. The mode of visual observation improved the applicability and usability of our MIRA-CRISPR-Cas13a-MPXV system in resource-limited areas.

In this study, we chose the highly conserved *F3L* gene of MPXV as the target gene for detection. Based on the reference sequence (NC_063383.1), we designed specific MIRA primers to distinguish MPXV from the variola, vaccinia and cowpox viruses. As shown in Additional file 1: Fig. S1, the results of the 2% AGE suggested that the *F3R3* group presented the best amplification efficiency, achieving the brightest specific amplification while having no nonspecific amplification among the 16 pairs of primers. In addition, the size of the specific band in Additional file 1: Fig. S1 was 144 bp, which was consistent with the expected target size, indicating that the target DNA was successfully amplified by the MIRA assay.

**Fig. 2:**
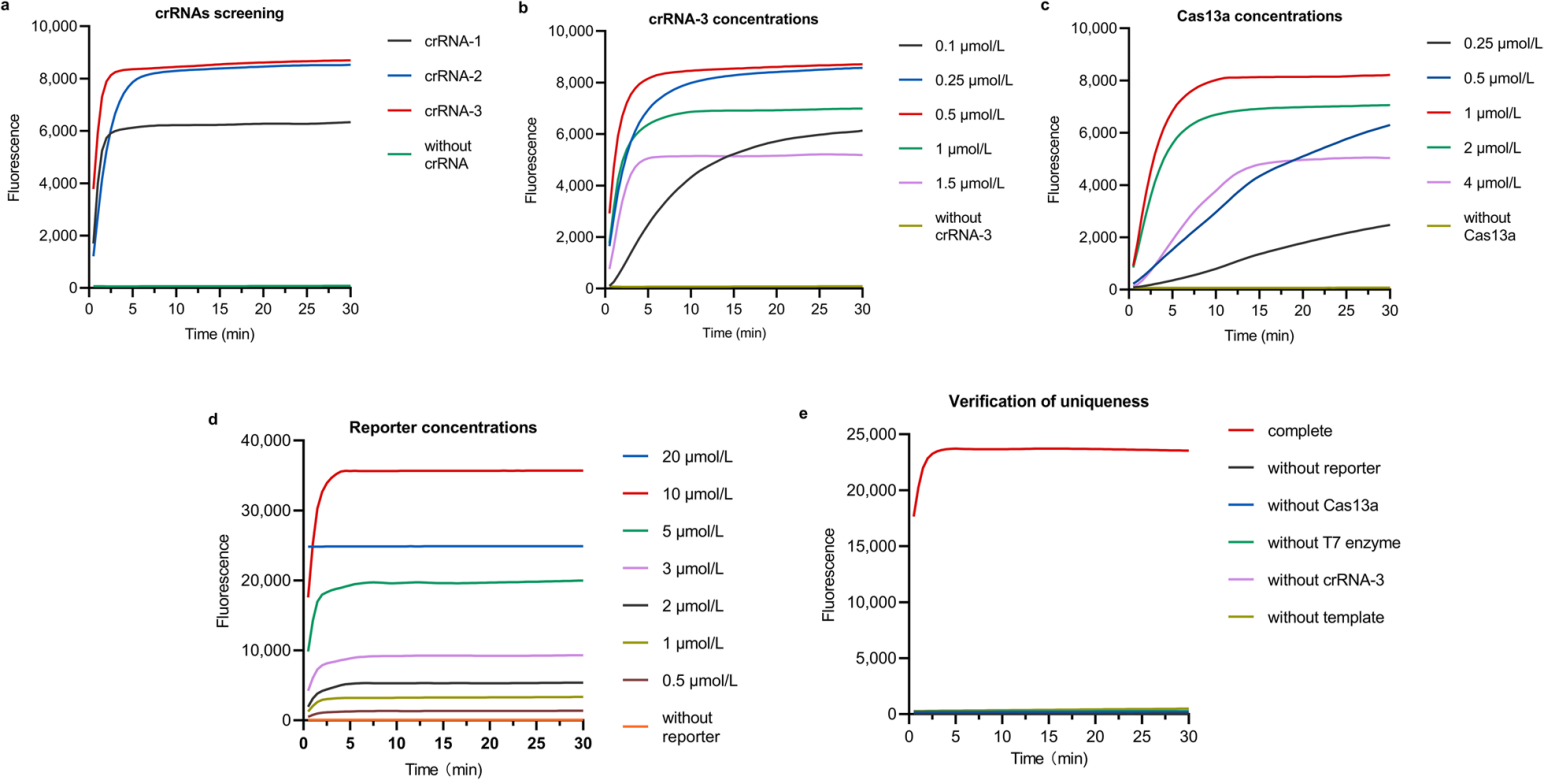
Optimization of CRISPR-Cas13a system reaction conditions. **a** Three candidate crRNAs screening, including crRNA-1, crRNA-2 and crRNA-3. **b** The fluorescence curve of crRNA-3 concentration optimization, including 0.1 µmol/L, 0.25 µmol/L, 0.5 µmol/L, 1 µmol/L and 1.5 µmol/L. **c** The fluorescence curve of LwaCas13a concentration optimization, including 0.25 µmol/L, 0.5 µmol/L, 1 µmol/L, 2 µmol/L and 4 µmol/L. **d** The fluorescence curve of reporter concentration optimization, including 1 µmol/L, 2 µmol/L, 3 µmol/L, 5 µmol/L, 10 µmol/L and 20 µmol/L. **e** Verification of the uniqueness of essential components of CRISPR-Cas13a system, including reporter, LwaCas13a, T7 enzyme, crRNA-3 and template

In the following CRISPR-Cas13a reaction, we designed three candidate crRNAs according to the results of the previous MIRA assay, and the results showed that all three candidate crRNAs could cut the reporter while crRNA-3 had the best effect, with the fastest reaction and the highest fluorescence intensity, as shown in Fig. 2a. Several essential components of this assay, including LwaCas13a, crRNA-3 and the reporter, were iteratively optimized and only one reagent was modified in each experiment to obtain the optimal analytical performance of our assay. As shown in Fig. 2b–d, the results indicated that 1 µmol/L LwaCas13a (2 µl), 0.5 µmol/L crRNA-3 (1 µl) and 10 µmol/L reporter (1 µl) generated the best performance. Furthermore, we added one fewer component to each reaction, including T7 RNA polymerase, LwaCas13a, crRNA, reporter, and MIRA products, to verify the indispensability of essential components of the reaction system. As shown in Fig. 2e, except for the reaction tube with complete components, other reaction tubes lacking a certain component did not produce any fluorescence, indicating that the above components were essential for the CRISPR-Cas13a reactions.

### Performances of the proposed MIRA-CRISPR-Cas13a-MPXV fluorescence assay

**Fig. 3:**
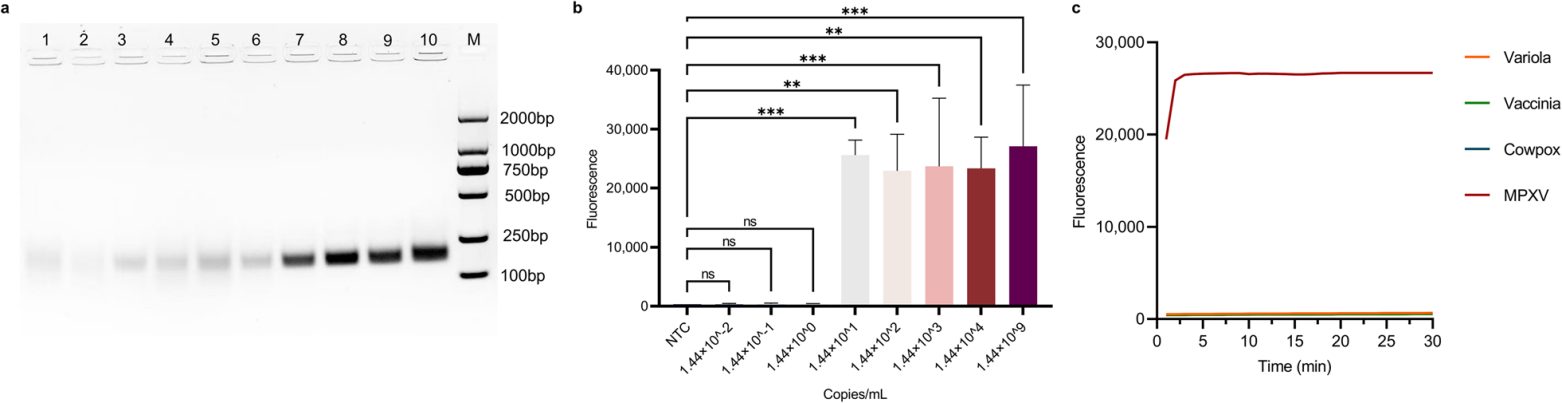
Performances of the proposed MIRA-CRISPR-Cas13a-MPXV fluorescence assay. **a** The gel image of MIRA assay sensitivity exploration via 2% agarose gel electrophoresis. Lane 1, no template control, use DNase/RNase-free water instead of the template; lane 2, blank, only reaction mixture; lane 3, 1.44 × 10^2^ copies/ml; lane 4, 1.44 × 10^3^ copies/ml; lane 5, 1.44 × 10^4^ copies/ml, lane 6, 1.44 × 10^5^ copies/ml; lane 7, 1.44 × 10^6^ copies/ml; lane 8, 1.44 × 10^7^ copies/ml; lane 9, 1.44 × 10^8^ copies/ml; lane 10, 1.44 × 10^12^ copies/ml; lane M, Molecular weight markers (FY2000 DNA marker, Yugong Biotech). 1.44 × 10^2^ copies/ml and above bands from weak to strong. **b** The analytical sensitivity of MIRA-CRISPR-Cas13a-MPXV system fluorescence assay. The plasmid serial dilution concentrations in the range of 1.44 × 10^–2^ copies/ml to 10^9^ copies/ml were subjected to MIRA-CRISPR-Cas13a-MPXV system fluorescence assay. ∗ , *P* < 0.05; ∗ ∗ , *P* < 0.01; ∗ ∗ ∗ , *P* < 0.001; ∗ ∗ ∗ ∗ , *P* < 0.0001; and ns, not significant. *P* < 0.05 indicated statistical significance. **c** Graph of the reaction process of CRISPR-Cas13a system detected three other OPVs plasmids compared with MPXV

The plasmid harbouring the synthesized *F3L* gene of MPXV was serially diluted to a range of 1.44 × 10^2^ to 10^12^ copies/ml, and the MIRA assay was performed. As shown in Fig. 3a, a weak band could be observed in the lane with 1.44 × 10^2^ copies/ml, and the bands became clearer as the plasmid concentration increased. Next, we diluted the plasmid to 1.44 × 10^–2^ copies/ml and performed the CRISPR-Cas13a assay. As shown in Fig. 3b, the fluorescence intensity of the assay with 1.44 × 10^1^ copies/ml and above was significantly different from that of the control group. A *P* value less than 0.05, as determined by one-way ANOVA, indicates statistical significance. Therefore, the LOD of MIRA-CRISPR-Cas13a fluorescence detection was considered 1.44 × 10^1^ copies/ml. We also synthesized homologous plasmids of other OPVs, including the *F3L* genes of the vaccinia, variola, and cowpox viruses, to test the specificity of the system. As shown in Fig. 3c, the detection system could not detect the *F3L* gene of three other OPVs, as no cleavage activity or accumulation of fluorescence signals were observed.

### Diagnostic performance of the MIRA-CRISPR-Cas13a system in clinical samples

**Fig. 4:**
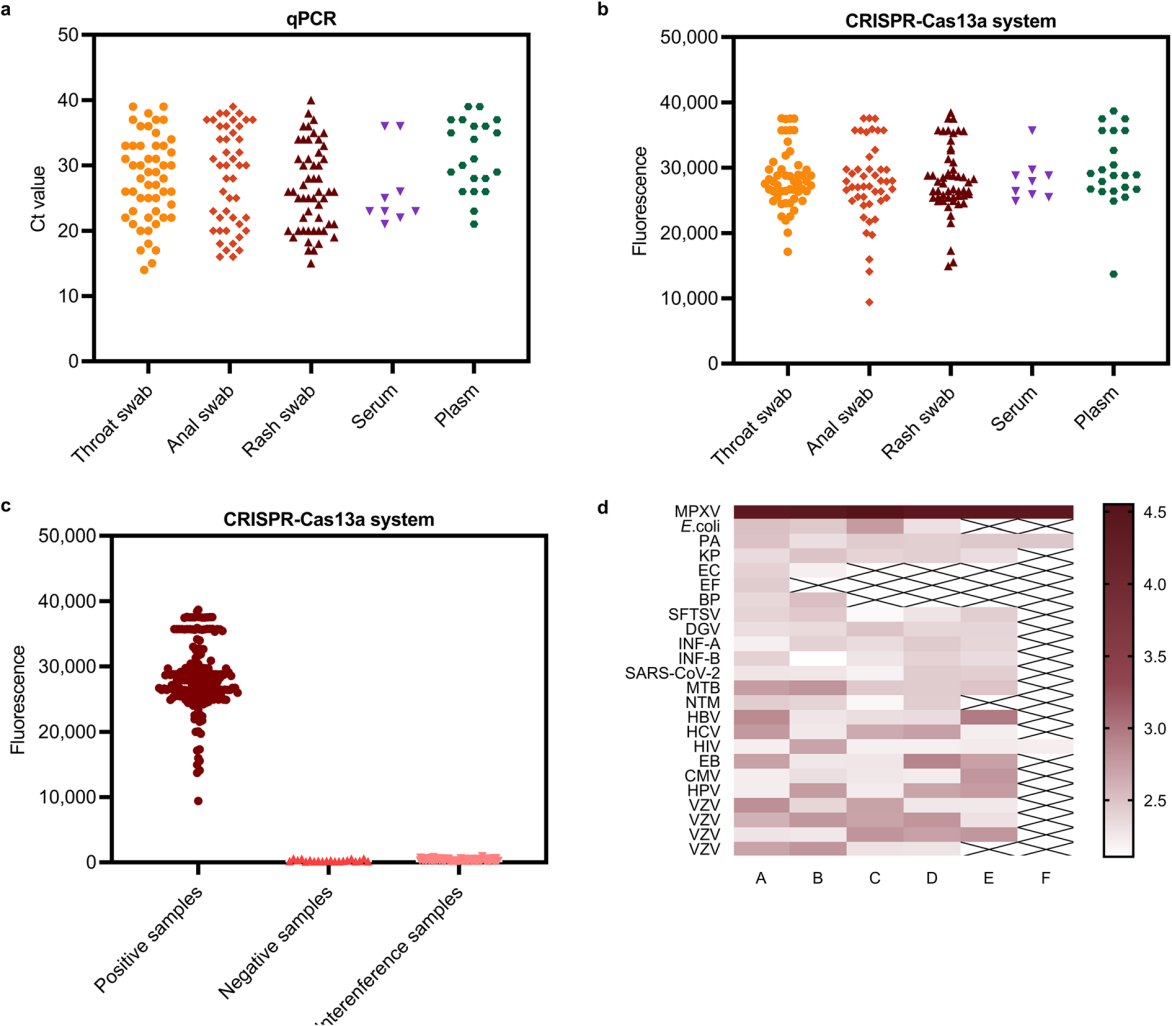
Clinical samples detected with qPCR and MIRA-CRISPR-Cas13a assay. **a** The corresponding Ct values for 179 clinical samples tested by qPCR assay. **b** The corresponding fluorescence intensities for 182 clinical samples tested by MIRA-CRISPR-Cas13a system. **c** The corresponding fluorescence intensities of MIRA-CRISPR-Cas13a assay for 182 positive samples, 20 negative samples and 104 interference samples. **d** Specificity evaluation of the proposed MIRA-CRISPR-Cas13a assay. A heat map corresponded to the log values of the interference sample results

After the analytical performance of the CRISPR-Cas13a system was evaluated, the feasibility of the proposed MPXV assay in clinical applications was further evaluated. A total of 202 clinical samples were tested using the gold-standard qPCR assay and then simultaneously tested using our CRISPR-Cas13a system to ensure the accuracy of the results. Notably, qPCR detected 179 positive samples and 23 negative samples, whereas the CRISPR-Cas13a assay detected 182 positive samples and 20 negative samples, as shown in Table 1 (detailed results for different sample types are shown in Additional file 1: Tables S7 and S8). Its kappa value was 0.994, indicating that our assay had good detection performance in real samples. Three samples were positive using CRISPR-Cas13a; however, the opposite results were obtained by qPCR. We traced the three samples and found that all three samples were from monkeypox positive patients and initially tested positive but presented high Ct values (34, 34 and 35 respectively). As shown in Fig. 4a, a total of 179 clinical samples tested positive by the qPCR assay, and the Ct values ranged from 14 to 40, including 51 rash fluid swabs, 52 oral swabs, 46 anal swabs, 9 serum samples and 21 plasma samples. Moreover, a total of 182 clinical samples that tested positive by the CRISPR-Cas13a system presented fluorescence values ranging from 9417.1 to 38,689.7, including 53 rash fluid swabs, 52 oral swabs, 46 anal swabs, 9 serum samples and 22 plasma samples, as shown in Fig. 4b. The results of the CRISPR-Cas13a system were essentially consistent with those of the qPCR assay for all clinical samples, indicating that the proposed CRISPR-Cas13a system for MPXV detection had competitive detection performance compared with the gold-standard qPCR assay, but presented higher detection performance, more concise operation, faster reaction speed, and stronger field deployment ability.

**Table 1:** Concordance between qPCR and our proposed MIRA-CRISPR-Cas13a system of MPXV DNA among real clinical samples

Methods		Results of qPCR		Total	Kappa
		Positive	Negative		
Results of MIRA-CRISPR-Cas13a system	Positive	179	3	182	0.994
	Negative	0	20	20	
	Total	179	23	202

A total of 104 interference samples were tested to confirm the specificity of the MIRA-CRISPR-Cas13a system in clinical samples. As shown in Fig. 4c, the fluorescence values of the other pathogen samples were significantly lower than those of the MPXV-positive samples, indicating that the above interference samples did not trigger false-positive reactions. As shown in Fig. 4d, a heatmap was drawn based on the results for the interference samples and the positive samples, and an obvious difference between the two could be visually observed. In summary, the above experiments suggested that the CRISPR-Cas13a system we proposed has strong anti-interference ability and can distinguish MPXV from other pathogens.

### MIRA-CRISPR-Cas13a-MPXV lateral flow strip assay for point-of-care testing

**Fig. 5:**
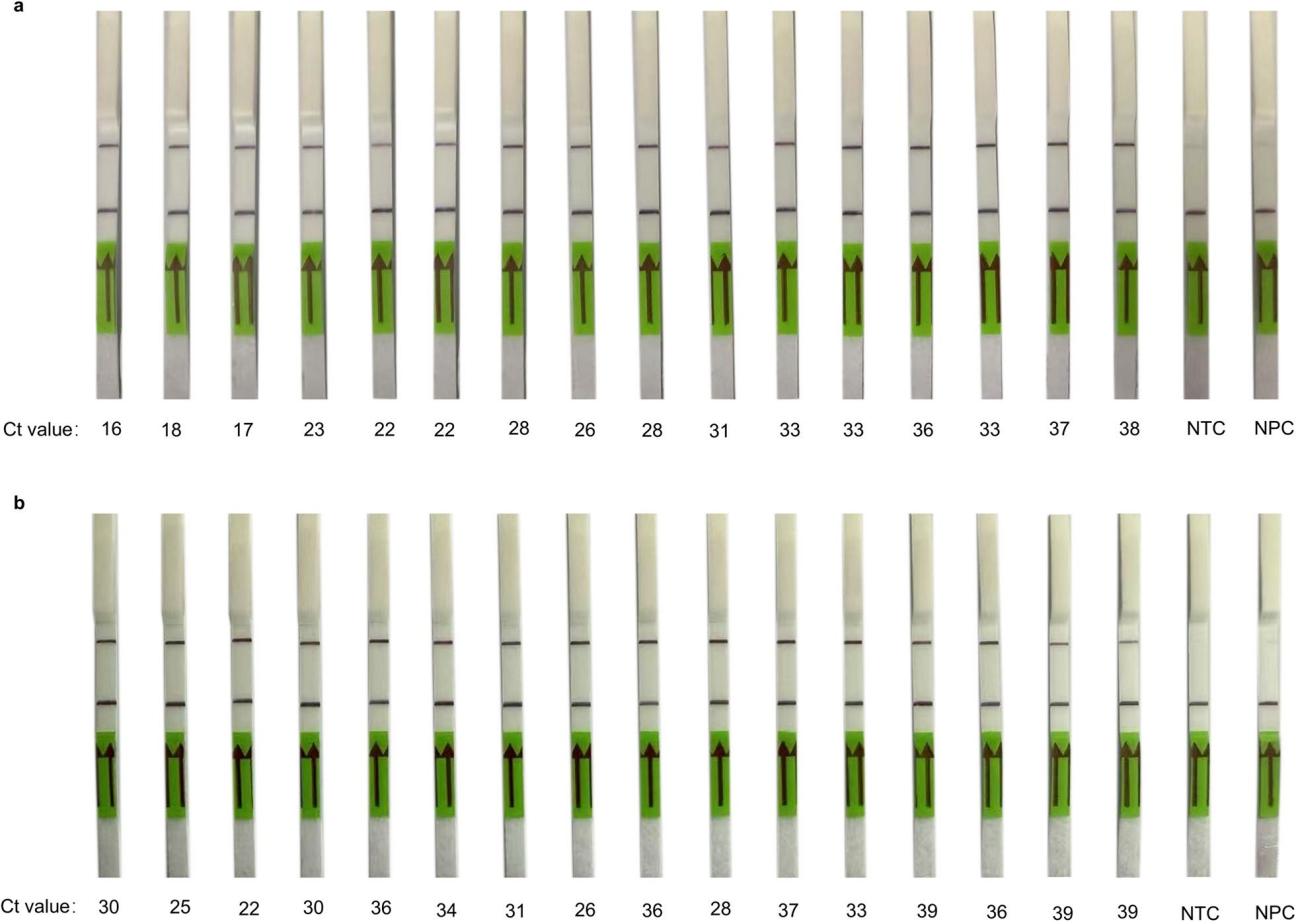
The clinical validation of MIRA-CRISPR-Cas13a system lateral flow strip assay. Results of lateral flow strip corresponded to multiple Ct values of clinical samples. *NTC* no MPXV DNA template control;* NPC* no MIRA product control. The number below each strip represents the Ct value of the corresponding clinical sample tested by the qPCR assay

Considering the application of point-of-care detection, we designed an FB reporter as a substitute for the FQ reporter and established a detection method using MIRA-CRISPR-Cas13a lateral flow strips. When the strips were added to the product of the CRISPR-Cas13a reaction treated with the buffer, the samples migrated forwards via chromatography, and the negative and positive samples presented different results. As shown in Fig. 5, the deletion of the DNA template in the negative group and blank group resulted in no Cas13a-activated amplicon or reporter cleavage; thus, only colour rendering in the quality control band occurred. The results of 32 clinically positive monkeypox samples with different Ct values showed that the strips were perfectly suited to our method and could be used to detect monkeypox samples at different concentrations.

### Development of a fluorescent PCR-based clade assay and its performance in clinical samples

The high sequence similarity between Clade I and Clade II MPXVs (> 99%) and other OPVs (> 90%) made the development of robust and clade specific target sequences challenging. In this study, we targeted the *F3L* gene in the conserved region to design general primers and probes while targeting the *C3L* gene to design specific primers and probes. Compared with Clade I, a deletion in the *C3L* gene was detected in Clade II, as shown in Additional file 1: Fig. S2. This deletion provided an alternative approach for us to design specific primers and probes to identify Clades I and II. As shown in Table 2, the results of the statistical analysis revealed that when the plasmid concentrations were 500 and 200 copies/ml, the positive detection rates of the corresponding target channels were ≥ 95%. However, when the plasmid concentrations were 150 and 100 copies/ml, the positive detection rates were less than 95%. As shown in Table 3, the results of the probit analysis revealed that the positive detection rates of the *F3L* gene, Clade I *C3L* gene and Clade II del*C3L* gene were ≥ 95%, with a concentration of approximately 200 copies/ml [95% confidence interval (*CI*) for the *F3L* gene: 173.528, 230.830; 95% *CI* for Clade I: 169.558, 228.834; 95% *CI* for Clade II: 159.150, 223.514]. The *F3L* gene, Clade I *C3L* I gene and Clade II del*C3L* gene were prepared at a concentration of 200 copies/ml and this analysis was repeated 20 times to verify the LOD of this assay. As shown in Table 4, the positive rate of each target channel was ≥ 95%, which indicated that 200 copies/ml as the LOD for the method was acceptable. As shown in Additional file 1: Table S9, the cross-examination of Clades I and II was repeated three times and no cross-activity was detected using our method. In addition, tests on three plasmids of the OPV species homologous to MPXV were negative, as shown in Additional file 1: Fig. S3a. In summary, the above results indicated that this assay could distinguish Clades I and II.

**Table 2:** Statistics on the positive rate of *F3L*, Clade I *C3L*, Clade II del*C3L* gene at each concentration

Concentration (copies/ml)	Channel	*F3L* gene plasmid	Channel	*C3L* Clade I gene plasmid	Channel	*C3L* Clade II gene plasmid
500	FAM	20/20	VIC	20/20	ROX	20/20
Positive rate		100%		100%		100%
200	FAM	19/20	VIC	20/20	ROX	20/20
Positive rate		95%		100%		100%
150	FAM	14/20	VIC	12/20	ROX	15/20
Positive rate		70%		60%		75%
100	FAM	2/20	VIC	3/20	ROX	5/20
Positive rate		10%		15%		25%

FAM: Carboxyfluorescein; VIC: 6-VIC phosphoramidite; ROX: Rhodamine X

**Table 3:** Probit analysis of LOD (95% detection)

LOD	*F3L*, 95% CI	Clade I *C3L*, 95% CI	Clade II del*C3L*, 95% CI
(copies/ml)	192.235 (173.528, 230.830)	188.352 (169.558, 228.834)	178.466 (159.150, 223.514)

LOD: Limit of detection

**Table 4:** The positive rate of 200 copies/mL of *F3L*, Clade I *C3L*, Clade II del*C3L* gene

Concentration (copies/ml)	Channel	*F3L* gene plasmid	Channel	Clade I *C3L* gene plasmid	Channel	Clade II del*C3L* gene plasmid
200	FAM	20/20	VIC	20/20	ROX	20/20
Positive rate		100%		100%		100%
200	FAM	20/20	VIC	20/20	ROX	20/20
Positive rate		100%		100%		100%
200	FAM	20/20	VIC	20/20	ROX	20/20
Positive rate		100%		100%		100%

FAM: Carboxyfluorescein; VIC: 6-VIC phosphoramidite; ROX: Rhodamine X

As shown in Table 5, through the testing 202 clinical MPXV samples, our proposed method identified 25 samples that were negative and 177 samples that were from Clade II (detailed information shown in Additional file 1: Table S10), which was consistent with the MPXV clade circulating in mainland China. Its kappa value was 0.994, indicating that the clade identification method had good performance. All 104 clinical interference samples tested negative, further demonstrating the high specificity of our approach. Representative curves amplified by our clade identification assay are shown in Additional file 1: Fig. S3b, c, d for positive, negative and interference clinic samples respectively. In general, the proposed clade identification assay has good detection ability and is easy to perform. Notably, we also verified the kappa consistency between the results of the clade identification assay and the MIRA-CRISPR-Cas13a-MPXV assay, and its kappa value was 0.994, indicating a good consistency between the two methods. As shown in Table 6, 177 clinical samples tested positive, and 20 clinical samples tested negative in both the MIRA-CRISPR-Cas13a-MPXV and clade identification assays. As shown in Table 5 and 6, three samples tested negative by the qPCR assay, but tested positive using our CRISPR-Cas13a system and clade identification assay. Five samples showed no clade identification but were positive in the qPCR and CRISPR-Cas13aassays. The eight samples were re-examined to eliminate the error caused by the test operation, and the results were consistent with the previous results.

**Table 5:** Concordance between qPCR and our proposed clade identification of MPXV DNA among real clinical samples

		Results of qPCR		Total	Kappa
		Positive	Negative		
Results of clade identification	Positive	174	3	177	0.994
	Negative	5	20	25	
	Total	179	23	202

**Table 6:** Concordance between MIRA-CRISPR-Cas13a-MPXV and clade identification of MPXV DNA among real clinical samples

		Results of MIRA-CRISPR-Cas13a-MPXV system		Total	Kappa
		Positive	Negative		
Results of clade identification	Positive	177	0	177	0.994
	Negative	5	20	25	
	Total	182	20	202	

## Discussion

The re-emergence and increasing incidence of MPXV is becoming an imminent threat to global health, and accelerating international public health measures and collaboration is imperative [31]. West Africa, Central Africa and other underdeveloped regions face diagnostic challenges related to the lack of connectivity of the nucleic acid amplification testing platform and cold chain requirements for sample preservation, which hinder case confirmation in these areas. The COVID-19 pandemic has demonstrated the need for affordable, rapid and precise diagnostic methods that can be deployed during POCT. Fluorescent qPCR and sequencing methods are the gold standard for diagnosing MPXV infection, but they are expensive and highly instrumentally demanding. These limitations have restricted their accessibility in resource-constrained areas where MPXV is prevalent, thereby negating their suitability for in-field epidemic surveillance applications. MIRA combined with CRISPR-Cas system mediated target recognition enables high detection sensitivity and specificity and provides an opportunity to implement low-complexity POCT for infectious diseases, including MPXV.

In this study, we aimed to develop rapid, sensitive, and specific tests for immediate detection that are economical, readily available, and easy to implement. We selected the highly conserved *F3L* gene to design the primers and ensure the specificity of this method. By screening the best primer group and corresponding crRNA that produced the best detection signal, our assay was applied to a large set of clinical samples and was compared with the gold-standard qPCR assay for consistency. A total of 202 clinical MPXV samples were tested and the results showed that our method had a better detection capability than the qPCR assay. Notably, three samples could not be detected by the qPCR assay (samples from confirmed MPXV positive patients and previously tested positive) but could be detected by the CRISPR-Cas13a system, suggesting that our method has increased reliability and practicality. Moreover, we also tested a range of interference clinical samples including but not limited to a variety of bacteria and respiratory and bloodborne viruses, to further verify the specificity in clinical samples, and the results showed that our method was highly specific. Notably, this analysis also included viruses with similar clinical manifestations, such as varicella zoster virus. Moreover, considering the application of this method at the point of care, we also studied the utilization of lateral flow test strips to interpret the results after studying the fluorescence detection method. Through the detection of 32 clinical samples, we found that lateral flow strips had a high detection sensitivity and could identify samples with a Ct value of 39 as positive. These advantages revealed that our assay could be combined with lateral flow strips to achieve a rapid, sensitive MPXV diagnosis at the point of care.

A previous study reported that the use of isothermal amplification before the CRISPR-Cas reaction could significantly improve the sensitivity of the detection system [32]. Thus, we adopted a combination of MIRA and CRISPR-Cas technology to achieve lower analytical sensitivity in this work. The MIRA reaction only requires a simple heating instrument, such as a water bath, to maintain the reaction at a constant temperature (37 °C), and the product can be expanded exponentially within 30 min. The operation of MIRA is similar to recombinase polymerase amplification (RPA), and both are isothermal amplification methods. However, compared with RPA, MIRA has a lower detection cost and a more stable and efficient enzyme detection system [33]. Most reported CRISPR-Cas methods use Cas12a for DNA detection because Cas12a can directly act on target DNA amplicons without additional transcription steps [30, 34]. Nevertheless, we still chose Cas13a over Cas12a in this study because it does not require a specific primary spacer adjacent motif, a feature that makes it more generic [35–37]. More importantly, Cas13a has been shown to have stronger collateral cleavage activity than Cas12a [38]. Furthermore, we also systematically optimized the concentrations of several important components, including LwaCas13a, crRNA, and the reporter, to increase the sensitivity of the reaction system. The results showed that 1 µmol/L Cas13a (2 µl), 0.5 µmol/L crRNA (1 µl) and 1 µmol/L reporter probe (1 µl) had the best response efficiency. By continuously diluting the synthesized plasmid, our method could detect as few as 14.4 copies/ml of the target gene. However, the qPCR method outlined in the China CDC’s Technical Guidelines for Monkeypox Prevention and Control generally reaches a detection limit of approximately 200 copies/ml. This also explains the inconsistent results of the three samples detected by our method and qPCR assay. In addition, this method was unable to obtain positive results for the *F3L* gene of the three synthetic OPV plasmids, indicating that the assay was sufficiently specific. The specific target recognition of crRNA and the signal amplification mechanism of CRISPR-Cas system provide a novel approach into the feasibility of multiplex detection.

Many published monkeypox assays rely on synthetic plasmids, pseudoviruses and simulated clinical samples, and the performance of these assays on clinical samples has not been evaluated [39–42]. For example, Zhao et al. reported that the combination of Cas12a with test strips could achieve a sensitivity of 10 copies/µl, but this result was not verified in real clinical samples [41]. Singh et al. exploited differences in single nucleotide polymorphisms to develop a Cas12a-based method that could detect MPXV at 1 copy/µl within 30 min. However, they did not validate this method in real samples [42]. Zhao et al. established a one-tube RPA-Cas12a system to detect and differentiate MPXV from other OPVs at a minimum concentration of 1–10 copies of viral DNA within 30 min. Nevertheless, the clinical samples used in their study were sourced from only one monkeypox patient, indicating an inadequacy in the diversity of samples [32]. In contrast, our method was able to detect MPXV in a range of different types of clinical samples, including rash blister fluid, throat swabs, anal swabs, serum, and plasma, which highlights the excellent performance of our analytical method in practical scenarios. Moreover, a systematic analysis of the Ct values of positive MPXV samples revealed that the viral load in MPXV patients' lesions was typically around a threshold of Ct 30 or lower, which is consistent with the findings of Vicente et al. [43]. Paran et al. reported a strong correlation between the amount of MPXV DNA and viral infectivity in clinical samples, suggesting that a threshold of Ct ≥ 35 (equivalent to ≤ 430 0 copies /ml) could be used to predict poor or noninfectious samples [44]. In our study, we were able to detect samples with a Ct of 35 or lower viral loads, indicating that our assay could detect MPXV in individuals during the infection period.

Currently, few studies on the identification of the monkeypox clade have been reported. Most of the published studies were generic assays. However, we further investigated clade detection after developing a general detection method. We designed primers and probes to differentiate Clades I and II by targeting regions with large differences. Notably, the primers and probes for Clade I were capable of detecting Clades Ia and Ib, and the primers and probes for Clade II were capable of detecting Clades IIa and IIb. The whole reaction process took only 40 min and was easy to perform. The sensitivity of this method could reach 200 copies/ml, and no cross-reaction was observed between the two clades. More importantly, we validated this method on various types of clinical samples. By testing 202 clinical MPXV samples and 104 interference samples, the results showed that our classification method could correctly identify the positive samples as Clade II. The clade could not be identified in five samples, probably because genetic mutations prevent the binding of the primers.

A limitation of our study was that the samples were obtained from one hospital during the MPXV outbreak in 2023 and were limited to Clade II. However, the primers and crRNA used in this study targeted the *F3L* gene, a noncoding region, that covers all MPXV clades. An additional limitation of our study was the two-tube step inspection, which could cause contamination during cap opening due to operator malpractice. However, this modularity allowed us to systematically optimize and exclude every component of detection at this stage of development. Future work will focus on exploring one-tube MIRA-CRISPR-Cas13a reactions and simple DNA extraction methods, which would also reduce the possibility of contamination. Another focus of our work is the feasibility of expanding this system for multiplex detection, such as simultaneous detection of MPXV and other OPVs.

## Conclusion

In conclusion, by utilizing the inherent collateral cleavage properties of the CRISPR-Cas13a system, we successfully developed a method capable of detecting the monkeypox *F3L* gene. Combined with MIRA, the assay achieved an analytical sensitivity of 14.4 copies/ml and did not react with the three other OPVs. By detecting 202 clinical MPXV samples and 104 interference samples, our method displayed high specificity and clinical applicability. We also developed a fluorescent PCR-based method targeting the *C3L* gene to distinguish Clades I and II, that could reach a sensitivity of 200 copies/ml and did not react with the three other OPVs. Similarly, during the validation of the abovementioned clinical samples, our method could correctly identify positive samples as Clade II. We provided a sensitive, specific, fast and easy to implement method for the detection of MPXV and its clades, which will contribute to monitoring the epidemiology of MPXV and controlling the spread of MPXV.

## Supplementary Information

Additional file 1

## Data Availability

All the data generated or analysed during this study are included in this published article and its Supplementary Information files.

## Abbreviations

MPXV: Monkeypox virus
MIRA: Multienzyme isothermal rapid amplification
CRISPR: Clustered regularly interspaced short palindromic repeats
qPCR: Quantitative PCR
OPV: *Orthopoxvirus*
POCT: Point-of-care testing
crRNA: CRISPR RNA
AGE: Agarose gel electrophoresis
SFTSV: Severe fever with thrombocytopenia syndrome virus
DFV: Dengue fever virus
INF-A: Influenza A virus
INF-B: Influenza B virus
HBV: Hepatitis B virus
HCV: Hepatitis C virus
HIV: Human immunodeficiency virus
EBV: Epstein‒Barr virus
CMV: Cytomegalovirus
HPV: Human papillomavirus
VZV: Varicella-zoster virus
KP: *Klebsiella pneumoniae*
PA: *Pseudomonas aeruginosa*
EC: *Enterobacter cloacae*
EF: *Enterococcus faecalis*
BP: *Bordetella pertussis*
MTB: *Mycobacterium tuberculosis*
NTM: Nontuberculous mycobacteria
Ct: Cycle threshold
FQ: FAM-BHQ1
FB: FITC-Biotin
LOD: Limit of detection
RPA: Recombinase polymerase amplification
SD: Standard deviation
CI: Confidence interval
