# Supplementary material for: An ultrasensitive and specific CRISPR-Cas13a-mediated point-of-care assay for monkeypox detection and PCR-based clade detection
Source: Infect Dis Poverty. 2025 Jun 23;14:56. doi: 10.1186/s40249-025-01325-5 (PMC12183853; doi:10.1186/s40249-025-01325-5)
Supplement: Supplementary file 1 — Additional file 1 [file 40249_2025_1325_MOESM1_ESM.docx]

**Supplementary Figures**

**Fig. S1** **Primers screening of MIRA.** The gel image of 16 pairs of MIRA primers screening via 2% agarose gel electrophoresis. Lane M, Molecular weight markers (FY2000 DNA marker, Yugong Biotech); lane 1, *F1R1*; lane 2, *F1R2*; lane 3, *F1R3*; lane 4, *F1R4*; lane 5, *F2R1*; lane 6, *F2R2*; lane 7, *F2R3*; lane 8, *F2R4*; lane 9, no template control; lane 10, positive control provided by MIRA kit; lane 11, *F3R1*; lane 12, *F3R2*; lane 13, *F3R3*; lane 14, *F3R4*; lane 15, *F4R1*; lane 16, *F4R2*; lane 17, *F4R3*; lane 18, *F4R4*; lane 19, no template control; lane 20, positive control provided by MIRA kit. *F3R3* group exhibited the brightest band among 16 pairs.


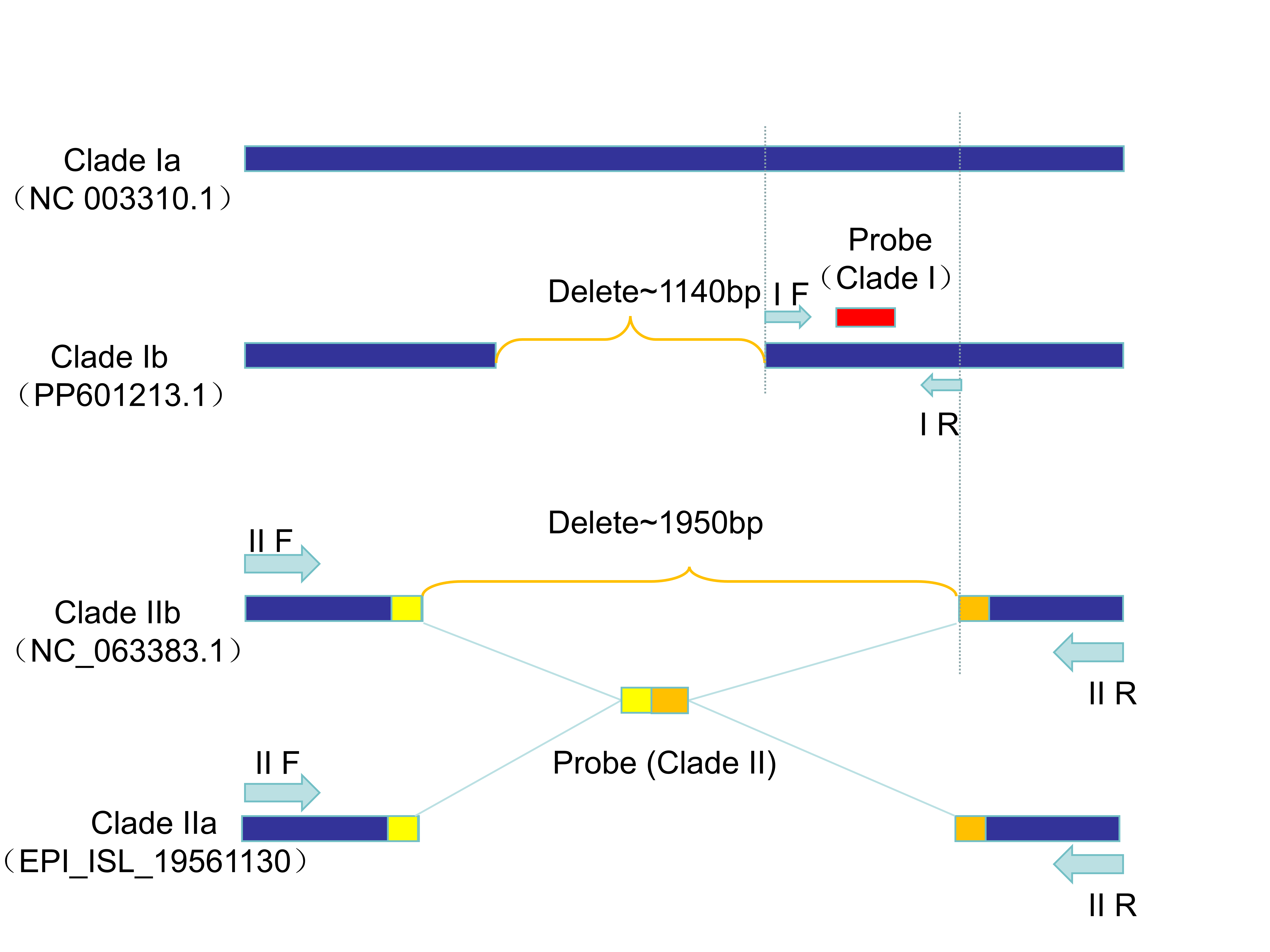


**Fig. S2** Primers of Clade Ia and Clade Ib cover the common region of both; Illustration of the Clade II primer probe target highlighting the probe design which spans up and downstream of the deleted region. I F: forward primer of Clade I; I R: reverse primer of Clade I. II F: forward primer of Clade II; II R: reverse primer of Clade II.


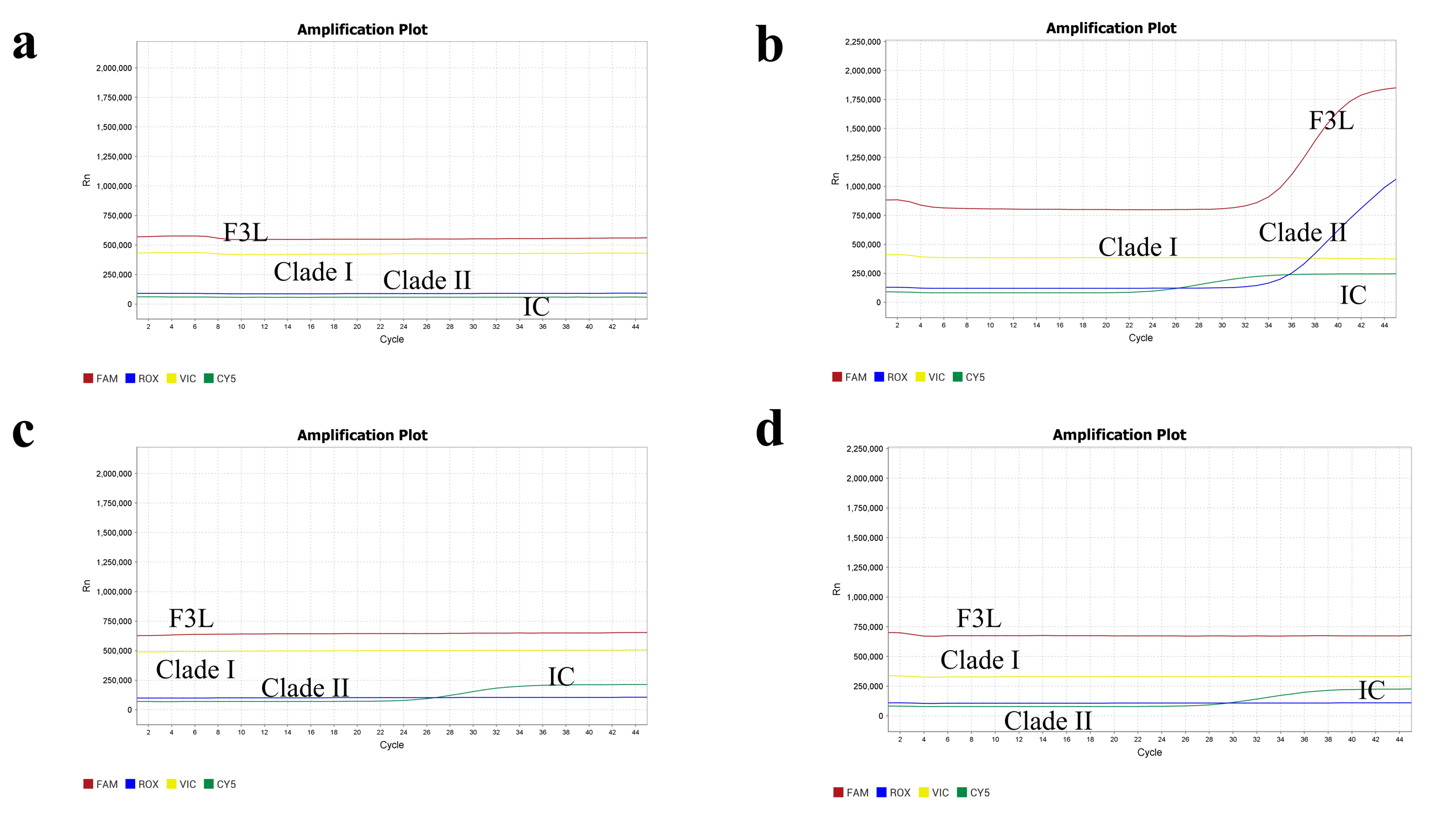


**Fig. S3 Graphs of samples tested by clade identification. a** Graph of the reaction process of clade identification detected three other *Orthopoxvirus* plasmids. **b** Graph of the reaction process of clade identification detected positive samples. **c** Graph of the reaction process of clade identification detected negative samples. **d** Graph of the reaction process of clade identification detected interference samples.

**Supplementary Tables**

**Table S1.** The sequences of primers, CRISPR crRNAs and reporters used in this study.

| Name | Sequence (5'-3') |
| --- | --- |
| MPXV-MIRA-F1 | 5'-GAAATTAATACGACTCACTATAGGGTCATCAAAAGAC  TTATGATCCTCTCTCATTGA-3' |
| MPXV-MIRA-F2 | 5'-GAAATTAATACGACTCACTATAGGGCTTATGATCCTCT  CTCATTGATTTTTCGCGGG-3' |
| MPXV-MIRA-F3 | 5'-GAAATTAATACGACTCACTATAGGGTGATCCTCTCTCA  TTGATTTTTCGCGGGATAC-3' |
| MPXV-MIRA-F4 | 5'-GAAATTAATACGACTCACTATAGGGTCTCATTGATTTT  TCGCGGGATACATCATCTA-3' |
| MPXV-MIRA-R1 | 5'-TATGATCTTCAACGTAGTGCTATGGTTTACAG-3' |
| MPXV-MIRA-R2 | 5'-TATGGTTTACAGCTCCAACGATACTCCTCCTC-3' |
| MPXV-MIRA-R3 | 5'-TCCAACGATACTCCTCCTCGTTGGTCTACGAC-3' |
| MPXV-MIRA-R4 | 5'-TACTCCTCCTCGTTGGTCTACGACAATGGATG-3' |
| MPXV-crRNA-1 | 5'-GAUUUAGACUACCCCAAAAACGAAGGGGACUAAAA  CUGGAUGCUGAUACACGGCCUACAGAUUC-3' |
| MPXV-crRNA-2 | 5'-GAUUUAGACUACCCCAAAAACGAAGGGGACUAAAA  CACAGAUUCUGAUGCUGAUGCUAUAAUAG-3' |
| MPXV-crRNA-3 | 5'-GAUUUAGACUACCCCAAAAACGAAGGGGACUAAAA  CUUCUGAUGCUGAUGCUAUAAUAGAUGAU-3' |
| Reporter FQ | 5' 6-FAM/rUrUrUrUrU/BHQ1-3' |
| Reporter FB | 5'-FITC-mArArUrGrGrCmAmArArUrGrGrCmA-Biotin-3' |

**Table S2.** The sequences of primers and probes of the monkeypox clades.

| Name | Sequence (5'-3') |
| --- | --- |
| F3L-F | 5'-CATCTATTATAGCATCAGCATCAGA-3' |
| F3L-R | 5'-GATACTCCTCCTCGTTGGTCTAC-3' |
| F3L-P | 5'-6-FAM-TGTAGGCCGTGTATCARCATCCATT-MGB-3' |
| Clade I-F | 5'-GCGCCTGAATATCTAGGAGTG-3' |
| Clade I-R | 5'-TCTATTTCAACGGGTATAGCAGAA-3' |
| Clade I-P | 5'-VIC-TCCATTTCGAAGTGCCGTGTTTCA-BHQ1-3' |
| Clade II-F | 5'-TCACTACGGACATAAACCATTGTA-3' |
| Clade II-R | 5'-CGTCGATGTCAGAAATAATCAACAA-3' |
| Clade II-P | 5'-ROX-CATTTTGGAAGTAAGTTCCTGGATCGG-BHQ2-3' |
| GAPDH-F | 5'-ATGCTGAGTGTACAAGCGTTTTCT-3' |
| GAPDH-R | 5'-CAGTCTGGGCACAAGCTTTG-3' |
| GAPDH-P | 5'-CY5-CTGAGCTAGGCAGCAGCAAGCATT-BHQ2-3' |

**Table S3.** The *F3L* gene sequences of synthesized monkeypox, cowpox, vaccinia, and variola plasmids.

| Virus | Sequence (5'-3') |
| --- | --- |
| Monkeypox | TCAGAATCTAATGATGACATAACTAAGAAGTTTATCTACAGCCAATTTAGCTGCATTATTTTTAGCATCTCGTTTAGATTTTCCATCTGCCTTATCGAATACTCTTCCGTCAATGTCTACACAGGCATAAAATGTAGGAGAGTTACTAGGCCCCACTGATTCAATACGAAAAGACCAATCTCTCCTAGTTATTTGACAGTACTCATTAATAACGGTGACAGGGTTAACACCTTTCCAATAAATAATTTTTTTAACCGGAATAACATCATCAAAAGACTTATGATCCTCTCTCATTGATTTTTCGCGGGATACATCATCTATTATAGCATCAGCATCAGAATCTGTAGGCCGTGTATCAGCATCCATTGTCGTAGACCAACGAGGAGGAGTATCGTTGGAGCTGTAAACCATAGCACTACGTTGAAGATCATACAGAGCTTTATTAACTTCTCGCTTCTCCAT |
| Cowpox | TCAGAATCTAATGATGACGTACCCAAGAAGTTTATCTACAGCCAATTTAGCTGCATTATTTTTAGCATCTCGTTTAGATTTTCCATCGGCCTTATCGAATACTCTTCCATCGATGTCTACACAGGCATAAAATGTAGGAGAGTTACTAGGTCCCACTGATTCAATACGAAAAGACCAATCTCTCTTAGTTATTTGGCAGTACTCATTAATAATGGTGACAGGGTTAGCATCTTTCCAATCAATAATTTTTTTGGCAGGAATAACATCATCAAAAGACTTATGATCCTCTCTCATTGATTTTTCGCGGGATACATCATCTATTATGACGTCAGCCATAACATCAGCATCCGTCTTATCCGCCTCCGTTGTCATAAACCAACGAGGAGGAATATCGTCGGAGCTGTACACCATATCACTACGTTGAAGATCGTACAGAGCTTTATTAACTTCTCGCTTCTCCATATTAAGTTGTCTAGTTAGTTGTGCAGCAGTAGCTCCTTCGATTCCAATGGTTTTAATAGCCTCACACACAATCTCTGCGTCAGAACGTTCGTCGATATAGATTTTAGACAT |
| Vaccinia | TTATTTACCATCCCATATATTCCATGAATAAGTGTGATGATTGTACACTTCTATAGTATCTATATACGATTCACGATAAAATCCTCCTATCAATAGCAGTTTATTATCCACTATGATCAATTCTGGATTATCCCTCGGATAAATAGGATCATCTATCAGAGTCCATGTATTGCTGGATTCACAATAAAATTCCGCATTTCTACCAACCAAGAATAACCTTCTACCGAACACTAACGCGCATGATTTATAATGAGGATAATAAGTGGATGGTCCAAACTGCCACTGATCATGATTGGGTAGCAAATATTCTGTAGTTGTATCAGTTTCAGAATGTCCTCCCATTACGTATATAACATTGTTTATAGATGCCACTGCTGGATTACATCTAGGTTTCAGAAGACTCGGCATATTAACCCAAGCAGCATCCCCGTGGAACCAACGCTCAACAGA TGTGGGATTTGGTAGACCTCCTACTACGTATAATTTATTGTTAGCGGGTATCCCGCTAGCATACAGTCTGGGGCTATTCATCGGAGGAATTGGAATCCAATTGTTTGATATATAATTTACAGCTATAGCATTGTTATGTATTTCATTGTTCATCCATCCACCGATGAGATATACTACTTCTCCAACATGAGTACTTGTACACATATGGAATATATCTATAATTTGATCCATGTTCATAGGATACTCTATGAATGGATACTTGTATGATTTGCGTGGTTGTTTATCACAATGAAATATTTTGGTACAGTCTAGTATCCATTTTACATTATTTATACCTCTGGGAGAAAGATAATTTGACCTGATTACATTTTTGATAAGGAGTAGCAGATTTCCTAATTTATTTCTTCGCTTTATATACCACTTAATGACAAAATCAACTACATAATCCTCATCTGGAACATTTAGTTCATCGCTTTCTAGAATAAGTTTCATAGATAGATAATCAAAATTGTCTATGATGTCATCTTCCAGTTCCAAAAAGTGTTTGGCAATAAAGTTTTTAGTATGACATAAGAGATTGGATAGTCCGTATTCTATACCCATCATGTAACACTCGACACAATATTCCTTTCTAAAATCTCGTAAGATAAAGTTTATACAAGTGTAGATGATAAATTCTACAGAGGTTAATATAGAAGCACGTAATAAATTGACGACGTTATGACTATCTATATATACCTTTCCAGTATATGAGTAAATAACTATAGAAGTTAAACTGTGAATGTCAAGGTCTAGACAAACCCTTGTAACTGGATCTTTATTTTTCGTGTATTTTTGACGTAAATGTGTGCGAAAGTAAGGAGATAACTTTTTCAATATCGTAGAATTGACTATTATATTGCCACCTATAGCATCAATAATTGTTTTGAATTTCTTAGTCATAGACAATGCTAATATATTCTTACAGTACACAGTATTAACAAATATCGGCAT |
| Variola | TCAATTCTGGATTATCCCTTGATAAATAGTATCATCTATCAGAGACCATGTATTGCTGTATTTGTAATAAAATTTAGCATTTCTACCAACCAAGAATAACCTTCTACCGAACACTAACGCGCATGATTTATAATGAGGATAATAAGTTGACGGTCCAAACTGCCACTGATCATGATTGGGTAGCAAATATTCTGTAGTTGTATCCGTTTCAGAATGTCCTCCTATTACGTACATAACATTGTTTATGGATGCCATTGCTGGATTACATCTAGGTTTCAAAAGACTTGGCATATTAACCCAAGCAGCATTCCCGTGGAACCAACGCTCAACAGATGTGGGATTTGGTAGACCTCCTACTACGTATAATTTATTGTTAGCGGGTATCCCGCTAGCATACAGTTTGGGGCTATTCATCGGAGGAATTGGAATCCAATTGTTTGATATATAATTTACCGCTATAGCATTGTTATGTATTTCATTGTTCAT |

**Table S4.** The sequences of synthesized MPXV F3L, Clade I *C3L*, Clade II del*C3L*, and GAPDH.

| Name | Sequence (5'-3') |
| --- | --- |
| MPXV F3L | AGATTTTCCATCTGCCTTATCGAATACTCTTCCGTCAATGTCTACACAGGCATAAAATGTAGGAGAGTTACTAGGCCCCACTGATTCAATACGAAAAGACCAATCTCTCCTAGTTATTTGGCAGTACTCATTAATAACGGTGACAGGGTTAACACCTTTCCAATAAATAATTTTTTTAACCGGAATAACATCATCAAAAGACTTATTATCCTCTCTCATTGATTTTTCGCGGGATACATCATCTATTATAGCATCAGCATCAGAATCTGTAGGCCGTGTATCAGCATCCATTGTCGTAGACCAACGAGGAGGAGTATCGTCGGAACTGTACACCATAGTACTACGTTGAAGATCATACAGAGCTTTATTAACTTCTCGCTTCTCCATTTAATATCTCTATCGTCCATATAAAATGGATTACTTAATGGATTGGCAAACCGTAACATACCGTTAGATAACTCTGCTCCATTTAGTACCGATTCTAGATACAAGATCATTCTACGTCCTATGGATGTGCAACTCTTAGCCGAAGCGTATGAGTATAGAGCACTATTTCTAAATCCCATCAGACCATATACTGAGTTGGCTACGATCTTGTACGTATACTGCATGGAATCATAGATGGCCTTTTCAGTTGAACTGGTAGCCTGTTTTAGCATCTTTTTATATCTGGCTCTCTCTGCCAAAAATGTTCTTAATAGTCT |
| Clade I *C3L* | CATGTATTTCCTGGAGAGCAAGTAGATGATGAGGAACCAGATAGTTTATATCCATACTTGCACTTAAAGTCTACATTGTAGTTGTATGAGTGTATGATCTTTTAAGCCGCTAGAAGTTTTCCGTTTGATATAGGATGTGGACATTTAACAATCTGACACGTGGGTGGATTGGACCATTCTCCTCCTGAACACATGACACCAGAGTTACCAATCAACGAATATCCACTATTGCAACTATAAGTTACAATGCTCCCATCGATATAAAAATCCTCGTATCCGTTATGTCTTCCGTTGGATATAGATGGAGGTGATTGGCATTTAACAGATTCGCAAATAGGTGCCTCAGGATTCCATACCATAGATCCAGTAGATCCTAATTCACAATACGATTTAGATTCACCGATCAAATGATATCCGCTATTACAAGAGTACGTTATACTAGAGCCAAAGTCTACTCCGCCAATATCAAGTTGGCCATTATCGATATCTCGAGGCGATGGGCATCTCCGTTTAATACATTGATTAAAGAGTGTCCATCCGGTACCGGTACATTTAGCATATATGGGTCCCATTTTTTGCTTTCTGTATCCAGGTAGACATAGATATTCTATAGTGTCTCCTATGTTGTAATTAGCATCAGTCTCTACACTATTCTTAAATTTCATATTAATGGGGCGTGACGGAATAGTACAGTATGATAGAACACATCCTATTCCCAACAATGTCAGGAACGTCACGCTCTCCACCTTCATATTTATTTATCCGTAAAATGTTATCCTGGACATCGTACAAATAATAAAAAGCCCATATATATGTTCGCTATTGTAGAAATTGTTTTTCACAGTTGCTCAAAAACAATGGCAGTGACTTATGAGTTAGTTACACTTTGGAGTCTCATCTTTAGTAAACATATCATAATATTCGATATTACGAGTTGACATATCGAACAAATTCCAAGTATTTGATTTTGGATAATATTCGTATTTTGCATCTGCTATAATTAAGATATAATCACCACAAGAACACACGAACGTCTTTCCTACATGGTTAAAGTACATGTACAATTCTATCCATTTGTCTTCCTTAACTATATATTTGTATAGATAATTACGAGTCTCATGAGTAATTCCAGTAATTGCATAGATGTCACCATCGTATTCTACAGCATAAACTATACTATGACGTCTAGGCATGGGAGACTTTTTTATCCAACGATTTTTAGTGAAACATTCCACATCGTTTAATACTACATATTTCTCATAGTGGTATAAACTCCACCCATTACATATATATCATCGTTTACGAATACTGATGCGCCTGAATATCTAGGAGTGATTAAGTTTGGAAGTCTTTTCCATTTCGAAGTGCCGTGTTTCAAATATTCTGCTATACCCGTTGAAATAGAAAATTCTAATCCTCCTATTACATATAACTTTCCATCGTTAACACAAGTACTAACTTCTGATTTTAACGACGACATATTAGTAACCGTTTTCCATT |
| Clade II del*C3L* | TATATTTTATGAAAGATATATCACTGCTCACCTCTATATTTCGTACATTTTTAAACTGTTTGTATAATATCTCTCTAATACAATCAGATATATCTATTGTGTCGGTAGACGATACCGTTACATTTGAATTAATGGTGTTCCATTTTACAACTTTTAACAAGTTGACCAATTCATTTCTAATAGTATCAAACTCTCCATGATTAAATATTTTAATAGTATCCATTTTATATCACTACGGACATAAACCATTGTATATTTTTTATGTTTATTAGTGTACACATTTTGGAAGTAAGTTCCTGGATCGGATGTCACCGCAGTAATATTGTTGATTATTTCTGACATCGACGTATTATATAGTTTTTTAATTCCATATCTTTTAGAAAAGTTAAACATCCTTATACAATTTGTGGAATTAATATTATGAATCATGGTTTTTACACATAGATCTATTACAGGCGGAACATCAATTATTATGGCAGCAACTAGTATCATTTCTACATTGTTTATGGTGATGTTTATCTTCTTCCAGCGCATATAGTCTAATATCGATTCAAACGCGTGATAGTTTATACCATTCAATATAATCGCT |
| GAPDH | CATGCACTTACCTGTGCTCCCACTCCTGATTTCTGGAAAAGAGCTAGGAAGGACAGGCAACTTGGCAAATCAAAGCCCTGGGACTAGGGGGTTAAAATACAGCTTCCCCTCTTCCCACCCGCCCCAGTCTCTGTCCCTTTTGTAGGAGGGACTTAGAGAAGGGGTGGGCTTGCCCTGTCCAGTTAATTTCTGACCTTTACTCCTGCCCTTTGAGTTTGATGATGCTGAGTGTACAAGCGTTTTCTCCCTAAAGGGTGCAGCTGAGCTAGGCAGCAGCAAGCATTCCTGGGGTGGCATAGTGGGGTGGTGAATACCATGTACAAAGCTTGTGCCCAGACTGTGGGTGGCAGTGCCCACATGGCCGCTTCTCCTGGAAGGGCTTCGTATGACTGGGGGTGTTGGGCAGCCCTGGAGCCTTCAGTTGCAGCCATGCCTTAAGCCAGGCCAGCCTGGCAGGGAAGCTCAAGGGAGATAAAATTCAACCTCTTGGGCCCTCCTGGGGGTAAGGAGATGCTGCATTCGCCCTCTTAATGG |

**Table S5.** Total 202 Clinical monkeypox samples information and corresponding Ct values from qPCR assay.

| **ID** | **Sample type** | **Viral Ct** | **ID** | **Sample type** | **Viral Ct** |
| --- | --- | --- | --- | --- | --- |
| 1 | Rash fluid swab | 34 | 102 | Throat swab | 36 |
| 2 | Throat swab | 34 | 103 | Anal swab | 18 |
| 3 | Anal swab | 35 | 104 | Throat swab | 28 |
| 4 | Rash fluid swab | 28 | 105 | Rash fluid swab | 19 |
| 5 | Rash fluid swab | - | 106 | Anal swab | 36 |
| 6 | Rash fluid swab | 34 | 107 | Rash fluid swab | 36 |
| 7 | Anal swab | 32 | 108 | Rash fluid swab | 28 |
| 8 | Rash fluid swab | 26 | 109 | Rash fluid swab | 37 |
| 9 | Rash fluid swab | 33 | 110 | Throat swab | 37 |
| 10 | Throat swab | 20 | 111 | Throat swab | 29 |
| 11 | Anal swab | 36 | 112 | Rash fluid swab | 22 |
| 12 | Rash fluid swab | 26 | 113 | Throat swab | 30 |
| 13 | Anal swab | 34 | 114 | Anal swab | 31 |
| 14 | Rash fluid swab | 20 | 115 | Throat swab | 17 |
| 15 | Rash fluid swab | 20 | 116 | Anal swab | 30 |
| 16 | Anal swab | 22 | 117 | Throat swab | 33 |
| 17 | Throat swab | 29 | 118 | Rash fluid swab | 25 |
| 18 | Rash fluid swab | 27 | 119 | Rash fluid swab | 21 |
| 19 | Anal swab | 16 | 120 | Throat swab | 33 |
| 20 | Rash fluid swab | 35 | 121 | Throat swab | 20 |
| 21 | Anal swab | 37 | 122 | Throat swab | 24 |
| 22 | Throat swab | 33 | 123 | Anal swab | 30 |
| 23 | Rash fluid swab | 32 | 124 | Throat swab | 18 |
| 24 | Anal swab | 26 | 125 | Rash fluid swab | 38 |
| 25 | Rash fluid swab | 31 | 126 | Rash fluid swab | - |
| 26 | Anal swab | 32 | 127 | Throat swab | 37 |
| 27 | Rash fluid swab | 30 | 128 | Anal swab | 38 |
| 28 | Throat swab | 30 | 129 | Throat swab | 26 |
| 29 | Anal swab | 16 | 130 | Rash fluid swab | 20 |
| 30 | Rash fluid swab | 34 | 131 | Throat swab | 37 |
| 31 | Throat swab | 39 | 132 | Throat swab | 22 |
| 32 | Anal swab | 38 | 133 | Anal swab | 17 |
| 33 | Rash fluid swab | 25 | 134 | Throat swab | 28 |
| 34 | Throat swab | 22 | 135 | Throat swab | 22 |
| 35 | Rash fluid swab | 23 | 136 | Rash fluid swab | 24 |
| 36 | Anal swab | 28 | 137 | Anal swab | 37 |
| 37 | Anal swab | 28 | 138 | Rash fluid swab | 17 |
| 38 | Throat swab | 26 | 139 | Anal swab | 17 |
| 39 | Anal swab | 19 | 140 | Throat swab | 32 |
| 40 | Throat swab | 23 | 141 | Anal swab | 38 |
| 41 | Throat swab | 14 | 142 | Anal swab | 23 |
| 42 | Anal swab | 25 | 143 | Rash fluid swab | 21 |
| 43 | Rash fluid swab | 20 | 144 | Plasm | 23 |
| 44 | Throat swab | 27 | 145 | Serum | 21 |
| 45 | Rash fluid swab | 22 | 146 | Serum | 22 |
| 46 | Anal swab | 25 | 147 | Plasm | 21 |
| 47 | Rash fluid swab | 31 | 148 | Serum | 23 |
| 48 | Rash fluid swab | 25 | 149 | Serum | 23 |
| 49 | Anal swab | 34 | 150 | Serum | 23 |
| 50 | Rash fluid swab | 19 | 151 | Plasm | 31 |
| 51 | Throat swab | 38 | 152 | Plasm | 37 |
| 52 | Rash fluid swab | 15 | 153 | Plasm | 30 |
| 53 | Throat swab | 33 | 154 | Plasm | - |
| 54 | Throat swab | - | 155 | Serum | 36 |
| 55 | Rash fluid swab | 25 | 156 | Serum | 25 |
| 56 | Throat swab | 15 | 157 | Serum | 26 |
| 57 | Anal swab | 37 | 158 | Plasm | 36 |
| 58 | Anal swab | 39 | 159 | Plasm | 39 |
| 59 | Throat swab | 36 | 160 | Plasm | 37 |
| 60 | Rash fluid swab | 26 | 161 | Plasm | 35 |
| 61 | Anal swab | 34 | 162 | Plasm | 36 |
| 62 | Throat swab | 25 | 163 | Plasm | 34 |
| 63 | Anal swab | 36 | 164 | Plasm | 35 |
| 64 | Rash fluid swab | 20 | 165 | Plasm | - |
| 65 | Throat swab | 31 | 166 | Plasm | - |
| 66 | Anal swab | 18 | 167 | Plasm | - |
| 67 | Throat swab | 25 | 168 | Plasm | 28 |
| 68 | Throat swab | 21 | 169 | Plasm | 26 |
| 69 | Throat swab | 35 | 170 | Plasm | 39 |
| 70 | Anal swab | 30 | 171 | Serum | 36 |
| 71 | Anal swab | 23 | 172 | Plasm | 28 |
| 72 | Throat swab | 22 | 173 | Plasm | 26 |
| 73 | Rash fluid swab | 28 | 174 | Plasm | 29 |
| 74 | Throat swab | 23 | 175 | Plasm | 26 |
| 75 | Throat swab | 31 | 176 | Plasm | 29 |
| 76 | Anal swab | 37 | 177 | Plasm | 37 |
| 77 | Rash fluid swab | 26 | 178 | Plasm | - |
| 78 | Throat swab | 31 | 179 | Anal swab | 21 |
| 79 | Anal swab | 20 | 180 | Rash fluid swab | 30 |
| 80 | Anal swab | 20 | 181 | Throat swab | 25 |
| 81 | Rash fluid swab | 26 | 182 | Anal swab | 20 |
| 82 | Rash fluid swab | 30 | 183 | Throat swab | 30 |
| 83 | Throat swab | - | 184 | Anal swab | 20 |
| 84 | Anal swab | 31 | 185 | Rash fluid swab | 20 |
| 85 | Rash fluid swab | 35 | 186 | Throat swab | 21 |
| 86 | Throat swab | 33 | 187 | Throat swab | 39 |
| 87 | Throat swab | 27 | 188 | Throat swab | - |
| 88 | Rash fluid swab | 40 | 189 | Throat swab | 17 |
| 89 | Anal swab | 37 | 190 | Rash fluid swab | 18 |
| 90 | Rash fluid swab | 17 | 191 | Throat swab | - |
| 91 | Throat swab | 30 | 192 | Serum | - |
| 92 | Rash fluid swab | 25 | 193 | Rash fluid swab | - |
| 93 | Anal swab | 22 | 194 | Throat swab | - |
| 94 | Throat swab | 26 | 195 | Throat swab | - |
| 95 | Anal swab | 22 | 196 | Throat swab | - |
| 96 | Rash fluid swab | 34 | 197 | Rash fluid swab | - |
| 97 | Anal swab | 30 | 198 | Throat swab | - |
| 98 | Rash fluid swab | 36 | 199 | Plasm | - |
| 99 | Rash fluid swab | 31 | 200 | Plasm | - |
| 100 | Throat swab | 26 | 201 | Throat swab | - |
| 101 | Anal swab | - | 202 | Plasm | - |

**Table S6.** Information of interfering samples used in cross-reactivity

| **ID** | **Pathogens** | **Material** | **ID** | **Pathogens** | **Material** |
| --- | --- | --- | --- | --- | --- |
| 1  2 | *E. coli* | Culture | 53 | NTM | Culture |
| 2 | PA | Culture | 54 | NTM | Culture |
| 3 | *E. coli* | Culture | 55 | HBV | Serum |
| 4 | *E. coli* | Culture | 56 | HBV | Serum |
| 5 | *E. coli* | Culture | 57 | HBV | Serum |
| 6 | KP | Culture | 58 | HBV | Serum |
| 7 | KP | Culture | 59 | HBV | Serum |
| 8 | KP | Culture | 60 | HCV | Serum |
| 9 | KP | Culture | 61 | HCV | Serum |
| 10 | KP | Culture | 62 | HCV | Serum |
| 11 | PA | Culture | 63 | HCV | Serum |
| 12 | PA | Culture | 64 | HCV | Serum |
| 13 | PA | Culture | 65 | HIV | Plasma |
| 14 | PA | Culture | 66 | HIV | Plasma |
| 15 | PA | Culture | 67 | HIV | Plasma |
| 16 | EC | Culture | 68 | HIV | Plasma |
| 17 | EC | Culture | 69 | HIV | Plasma |
| 18 | EF | Culture | 70 | HIV | Plasma |
| 19 | BP | Throat swab | 71 | EBV | Plasma |
| 20 | BP | Throat swab | 72 | EBV | Plasma |
| 21 | SFTSV | Serum | 73 | EBV | Plasma |
| 22 | SFTSV | Serum | 74 | EBV | Plasma |
| 23 | SFTSV | Serum | 75 | EBV | Plasma |
| 24 | SFTSV | Serum | 76 | CMV | Plasma |
| 25 | SFTSV | Serum | 77 | CMV | Plasma |
| 26 | DFV | Serum | 78 | CMV | Plasma |
| 27 | DFV | Serum | 79 | CMV | Plasma |
| 28 | DFV | Serum | 80 | CMV | Plasma |
| 29 | DFV | Serum | 81 | HPV | Tissue |
| 30 | DFV | Serum | 82 | HPV | Tissue |
| 31 | INF-A | Throat swab | 83 | HPV | Tissue |
| 32 | INF-A | Throat swab | 84 | HPV | Tissue |
| 33 | INF-A | Throat swab | 85 | HPV | Tissue |
| 34 | INF-A | Throat swab | 86 | VZV | Plasma |
| 35 | INF-A | Throat swab | 87 | VZV | Plasma |
| 36 | INF-B | Throat swab | 88 | VZV | Plasma |
| 37 | INF-B | Throat swab | 89 | VZV | Plasma |
| 38 | INF-B | Throat swab | 90 | VZV | Plasma |
| 39 | INF-B | Throat swab | 91 | VZV | Plasma |
| 40 | INF-B | Throat swab | 92 | VZV | Plasma |
| 41 | SARS-CoV-2 | Throat swab | 93 | VZV | Plasma |
| 42 | SARS-CoV-2 | Throat swab | 94 | VZV | Plasma |
| 43 | SARS-CoV-2 | Throat swab | 95 | VZV | Plasma |
| 44 | SARS-CoV-2 | Throat swab | 96 | VZV | Plasma |
| 45 | SARS-CoV-2 | Throat swab | 97 | VZV | Plasma |
| 46 | MTB | Bronchoalveolar lavage fluid Num | 98 | VZV | Plasma |
| 47 | MTB | Bronchoalveolar lavage fluid | 99 | VZV | Plasma |
| 48 | MTB | Bronchoalveolar lavage fluid | 100 | VZV | Plasma |
| 49 | MTB | Bronchoalveolar lavage fluid | 101 | VZV | Plasma |
| 50 | MTB | Bronchoalveolar lavage fluid | 102 | VZV | Plasma |
| 51 | NTM | Culture | 103 | VZV | Plasma |
| 52 | NTM | Culture | 104 | VZV | Plasma |

*E. coli: Escherichia coli*; PA: *Pseudomonas aeruginosa;* KP: *Klebsiella pneumoniae*; EC: *Enterobacter cloacae*; EF: *Enterococcus faecalis*; BP: *Bordetella pertussis*; SFTSV: New Bunia virus; DFV: dengue fever virus; IFN-A: Influenza A virus; INF-B: Influenza B virus; MTB: *Mycobacterium* *tuberculosis*; NTM: Nontuberculous mycobacteria; HBV: Hepatitis B virus; HCV: Hepatitis C virus; HIV: Human immunodeficiency virus; EBV: Epstein‒Barr virus; CMV:  cytomegalovirus; HPV: human papillomavirus; VZV: Varicella-Zoster Virus.

**Table S7.** qPCR results of 202 clinical samples of different types.

|  | Rash fluid swab | Throat swab | Anal swab | Serum | Plasm | Total |
| --- | --- | --- | --- | --- | --- | --- |
| Positive | 51 | 52 | 46 | 9 | 21 | 179 |
| Negative | 4 | 9 | 1 | 1 | 8 | 23 |
| Total | 55 | 61 | 47 | 10 | 29 | 202 |

**Table S8.** MIRA-CRISPR-Cas13a-MPXV results of 202 clinical samples of different types.

|  | Rash fluid swab | Throat swab | Anal swab | Serum | Plasm | Total |
| --- | --- | --- | --- | --- | --- | --- |
| Positive | 53 | 52 | 46 | 9 | 22 | 182 |
| Negative | 2 | 9 | 1 | 1 | 7 | 20 |
| Total | 55 | 61 | 47 | 10 | 29 | 202 |

**Table S9.** Ct values of cross-examination of Clade I and Clade II tested by the developed fluorescent PCR-based clade assay.

| Clade I *C3L* gene | | Clade II del*C3L* gene | |
| --- | --- | --- | --- |
| VIC  (*C3L* I) | ROX  (*C3L* II) | VIC  (*C3L* I) | ROX  (*C3L* II) |
| 27 | - | - | 27 |
| 27 | - | - | 27 |
| 27 | - | - | 27 |

**Table S10.** Clade identification results of 202 clinical samples of different types.

|  | Rash fluid swab | Throat swab | Anal swab | Serum | Plasm | Total |
| --- | --- | --- | --- | --- | --- | --- |
| Positive | 52 | 52 | 44 | 9 | 20 | 177 |
| Negative | 3 | 9 | 3 | 1 | 9 | 25 |
| Total | 55 | 61 | 47 | 10 | 29 | 202 |
